# Design and implementation of a national public health surveillance system in Jordan

**DOI:** 10.1016/j.ijmedinf.2016.01.003

**Published:** 2016-04

**Authors:** Sami Adel Sheikhali, Mohammed Abdallat, Sultan Mabdalla, Bashir Al Qaseer, Rania Khorma, Mamunur Malik, Maria Cristina Profili, Gunnar Rø, John Haskew

**Affiliations:** a Ministry of Health, Jordan; b World Health Organization, Cairo, Egypt; c World Health Organization, Amman, Jordan; d University of Durham, UK

**Keywords:** Public health surveillance, Information and communication technology, International classification of disease, Clinical diagnostic algorithms

## Abstract

Understanding and improving the health status of communities depend on effective public health surveillance. Adoption of new technologies, standardised case definitions and clinical guidelines for accurate diagnosis, and access to timely and reliable data, remains a challenge for public health surveillance systems however and existing public health surveillance systems are often fragmented, disease specific, inconsistent and of poor quality. We describe the application of an enterprise architecture approach to the design, planning and implementation of a national public health surveillance system in Jordan. This enabled a well planned and collaboratively supported system to be built and implemented using consistent standards for data collection, management, reporting and use. The system is case-based and integrated and employs mobile information technology to aid collection of real-time, standardised data to inform and improve decision-making at different levels of the health system.

## 1 Introduction

Public health surveillance is the ongoing, systematic assessment of the health of a community [1] and a national public health surveillance system plays an important role in ensuring that reliable and timely health information is available to inform operational and strategic decision making at different levels of the health system. Despite its importance for evidence-based decisions, public health surveillance in many countries is weak, fragmented and often focused on disease-specific program areas. Surveillance programs often rely on multiple layers of reporting structure from the facility to central level, which can result in delays in release of data and substantial human and time resources may be spent on data cleaning and analysis before they can be released and interpreted correctly [2]. Data sharing may also be impeded if standardised approaches are not used for coding and formatting of data. For example, aggregated data may be collected and reported from disease registers for which there is no quality control over the case definition used. Furthermore, aggregated data can limit the ability to link epidemiological data to laboratory confirmation of diagnosis and the ability to undertake contact tracing and follow-up of suspected notifiable diseases.

Technological and analytical innovations within public health surveillance systems, including the use of information and communication technology, may help in enabling standardised data to be collected in real-time, and several advantages have been discussed that could help to inform and improve decision-making [3], [4]. Electronic information can be collected in a structured, coded manner and, if the tool is used within the consultation, skip-logic algorithms can be used to provide clinical decision support in support of diagnosis and management of disease [5]. Mobile information technology also enables other data sources, including population and mapping data, and other tools for data visualisation, including Google Maps [6], to be more readily integrated into the process of data collection, reporting and analysis [7], [8].

## 2 Development of a public health surveillance framework in Jordan

An innovative national program of public health surveillance is being implemented across Jordan, in partnership with the Ministry of Health and World Health Organization (WHO), that uses mobile tools and an online framework for collection, analysis and reporting of surveillance data. Following pilot project implementation between May to December 2014, national scale-up and implementation of the public health surveillance system in Jordan took place in a phased manner in April 2015. A total of 269 primary and secondary care facilities are currently included in the system, using 409 mobile tablets, across all twelve governorates of the country.

An enterprise architecture approach was applied to identify the key elements and relationships required to develop the national public health surveillance system in Jordan. This approach enabled important interrelationships to be identified and reduce subsequent risks of fragmentation, duplication and lack of interoperability. The approach also mitigated the application of information and communication technology in an unplanned and unstructured manner. Four domains defined the general model of enterprise architectal public health surveillance system, each of which are considered in the following sections.

### 2.1 Organisational architecture

The organisational architecture of the public health surveillance system defines the various business functions, process, governance, policy and resources required for its development and implementation. The Ministry of Health own and manage the system, and multiple departments including communicable disease, non-communicable disease, mental health, information technology and training were involved in its design and implementation. High level approval from the Minister of Health and Prime Minister was obtained to ensure appropriate policy and resources could be ensured for effective implementation. A number of consultative meetings with other stakeholders and partners in the health sector, including military, private sector, other UN agencies and NGOs, are ongoing who may play a role in any future scale-up and implementation beyond the national health system.

The system was initially implemented as a pilot across 50 health facilities between May to December 2014 in northern Jordan, during which time technical aspects of implementation could be tested and refined. This period also enabled extensive consultation to take place peripherally (among health care workers) and centrally (among management) within the Ministry of Health to better understand needs and functionality for the system. This afforded institutional learning and buy-in to take place across the various departments and levels of Ministry of Health.

The first phase of national scale-up within Ministry of Health facilities began in April 2015 and the system is now operational in 269 facilities at primary, secondary and tertiary care level. The Ministry of Health conducted all training for implementation of the system, which included facility-level health care workers, governorate level and central health management. Training is continuous among all levels of the health system and a total of 1738 health personnel and 48 managers have been trained to date in use of the system.

### 2.2 Data architecture

The data architecture of the public health surveillance system includes the data model, data dictionary and classification of standards and systems used. Mobile tablets are used by health workers within the consultation to provide case-based reporting of disease as well as to introduce electronic modules for prescribing, using the WHO Model List of Essential Medicines [9] and clinical diagnostic algorithms, including the integrated management of childhood illness (IMCI) [10] and WHO Mental Health Gap Action Programme (mhGAP) [11]. Disease information is coded in a structured manner according to the International Classification of Disease (ICD-10) [12].

Reported information is made available within one hour via an online framework, with support for analysis, mapping and reporting that is accessed at all levels of Ministry of Health. Essential health system indicators are also collected through the system as defined by the WHO framework for health information systems and core indicators in the Eastern Mediterranean Region [13].

### 2.3 Applications architecture

**Fig. 1:**
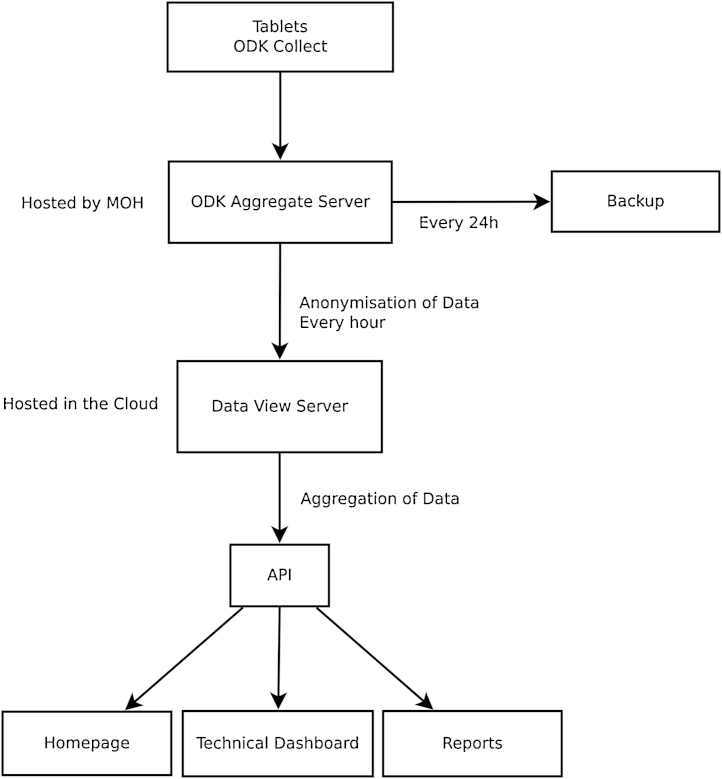
Diagram of public health surveillance system components and data flow.

The applications architecture of the public health surveillance system describes the modality of data collection, management, analysis and reporting as well as inter-operability of software applications and interfaces. A case-based reporting form, available in Arabic and English, was designed in extended mark-up language (XML) and uploaded to tablets for clinic consultations. The form is modular in design, collecting individual patient demographic information as well as relevant clinical, laboratory and prescribing information depending on user input. Each form is programmed for the Open Data Kit (ODK; www.opendatakit.org) [14] application and skip logic algorithms are used. Data are uploaded in real-time from the clinic tablets to a central server based at the Ministry of Health. Data are anonymised and aggregated over time and location using a custom designed application, based on a PostgreSQL (www.postgresql.org) database, a python (www.python.org) application programming interface (API) and a password protected HTML/Javascript website. This website is updated with aggregated and anonymised data every hour for access and use at all levels of the Ministry of Health. The custom-designed website displays demographics and proportional disease morbidity at directorate, district and health centre level and displays data using tables, charts and maps. Fig. 1 describes the public health surveillance system components and data flow.

### 2.4 Technical architecture

The technical architecture of the public health surveillance system describes systems infrastructure and hardware used in its development and implementation. The system was built using free, open source software and is implemented as a cloud-based model, rather than a local clinic model, which removes the need for local clinic infrastructure. In using the term “cloud-based” we refer to the fact that the server and data are hosted centrally by the Ministry of Health and not by the individual clinic.

This approach provides a potentially more cost-effective solution to implementation (removing the need for local security, network and computer hardware in each clinic) and also enhances data access and sharing at different levels of the health system. The estimated cost of national implementation across 269 sites, and using 409 tablets, was approximately USD$ 94,000, or USD$ 227 per reporting unit (tablet), which includes the unit cost of tablets, connection costs and training expenses. Subsequent support and system costs are approximately USD$ 40,000 per year, or USD$ 96 per reporting unit (tablet) per year.

The system uses a secure network, provided by a mobile phone operator in Jordan, to which the server and clinic tablets connected exclusively via a Hypertext Transfer Protocol Secure (HTTPS) connection. Android-based Lenovo IdeaTab 7″ A3000 and A3500 tablets are used in each health facility, with built-in mobile data connection, and are locked so users cannot use the device for any other function. Only tablets using SIM cards registered by the project can access the server. Fig. 1 describes the public health surveillance system components and data flow.

## 3 Conclusion

An enterprise architecture approach was applied to identify the key elements and relationships required to develop a national system of public health surveillance in Jordan and to inform the introduction of technological innovation. A number of innovative principles of public health surveillance are introduced, including the use of cloud-based, electronic data collection and management and the use of case-based reporting within the consultation. Several advantages of these features are postulated including the use of clinical decision support for disease diagnosis and management, standardisation and coding of data, provision of timely and reliable analysis and reporting and cost-effectiveness of implementation.

An evaluation of perceptions of use of the system, as well as descriptive epidemiological analysis of data collected by the system will be presented in separate papers. Further work is encouraged to compare the cloud-based public health surveillance model with traditional public health surveillance systems, including impact on cost-effectiveness, data completeness and data sharing and to consider the scalability of cloud-based models in settings where data network infrastructure is functional. Further studies are also encouraged to explore other applications of electronic surveillance, including the incorporation of clinical decision support to improve the quality of clinical care.

## Authors’ contributions

All authors contributed to the paper. JH conceived and designed the study. JH, GR, RK, SS designed and implemented the public health surveillance system. JH, GR, RK, SS, MCP, MM wrote the paper. All authors reviewed the final draft of the manuscript.

## Conflict of interest

No conflicts of interest are declared by the authors. No authors were paid for writing of the manuscript.

**Summary points**

What was already known on the topic:

• Existing public health surveillance systems are often fragmented, disease specific, inconsistent and of poor quality.
• Adoption of new technologies, standardised case definitions and clinical guidelines for accurate diagnosis, and access to timely and reliable data, remains a challenge for public health surveillance systems.
• Technological and analytical innovations within public health surveillance systems, including the use of information and communication technology, may help in enabling standardised data to be collected in real-time.

What this study added to our knowledge:

• An enterprise architecture approach was applied to identify the key elements and relationships required to develop the national public health surveillance system in Jordan.
• A number of innovative principles of public health surveillance are introduced, including the use of cloud-based, electronic data collection and management and the use of case-based reporting within the consultation.
• Several advantages of these features are postulated including the use of clinical decision support for disease diagnosis and management, standardisation and coding of data, provision of timely and reliable analysis and reporting and cost-effectiveness of implementation.
